# Editorial on the Special Issue Entitled “Recent Advances in Aerogels”

**DOI:** 10.3390/gels10030154

**Published:** 2024-02-20

**Authors:** Gabrijela Horvat, Uroš Maver

**Affiliations:** 1 Faculty of Chemistry and Chemical Engineering, University of Maribor, Smetanova ul. 17, SI-2000 Maribor, Slovenia; 2 Faculty of Medicine, University of Maribor, Taborska ul. 8, SI-2000 Maribor, Slovenia; uros.maver@um.si

Aerogels are unique solid materials that consist mainly of air and have an extremely low density, large open pores, and a large internal surface area. The term “aerogel” was first introduced by Kistler in 1931 [1] to designate gels in which the liquid was replaced with gas without collapsing the solid network. Aerogels are normally produced using the sol–gel process, followed by a drying step, of which supercritical drying is the most commonly used approach. Supercritical drying is the final and most critical step in the production of aerogels, where the original solvent is exchanged for CO_2_ in a supercritical state, preventing the presence of two phases (gaseous and liquid) and structural collapse [2]. Aerogels belong to highly innovative materials, and their scientific impact has increased to the point that the IUPAC identified them as a “Top Ten Emerging Technology in Chemistry in 2022” [3]. The speciality of aerogels is that they can be made from organic or inorganic materials; the only requirement is that the starting material can form a gel.

The field of aerogels has seen tremendous growth in recent years, leading to their extensive use in research and translation to real-life applications. Current related research mainly focuses on applying aerogels in developing drug delivery systems, tissue engineering, insulation materials, energy storage, and parts of buildings, to name a few. With the development of new routes of aerogel synthesis, some old characterisation methods are also being questioned, and new ones are being investigated. Novel materials from inorganic, organic, and hybrid aerogels with unique properties and functionalities are being prepared. The Special Issue, entitled “Recent Advances in Aerogels”, has been published in *Gels* (ISSN 2310-2861) under the section Gel Chemistry and Physics to cover all of the recent advancements related to aerogels.

A notable feature of this Special Issue is the diversity of topics covered, ranging from novel synthesis routes to practical aerogel applications in various fields. The submitted articles include one comprehensive review, providing a broad overview of pore structure determination for bioaerogels, along with eleven research articles presenting cutting-edge developments.

The variety of topics covered in the submissions, including the thermomechanical performance assessment of sustainable building insulating materials, the origin of the springback effect in ambient-pressure-dried silica aerogels by Fabian Zemke et al., and the preparation of bio-based aerogels for adsorption of organic dyes, showcases the broad spectrum of aerogel research. Fabian Zemke et al. reported that ambient-pressure-drying is crucial for preventing shrinkage or making it reversible in aerogel formation. The springback effect is a significant phenomenon during the ambient-pressure-drying of aerogels, and different surface modification agents can influence this behaviour. 

One of the research articles, authored by Marco Pedroso et al., addresses the integration of aerogel-based fibre-enhanced thermal renders in building walls. The optimised aerogel-based renders showed better performance due to lower acquisition costs. The regional climate significantly impacted optimum thickness, and cost-optimised formulations substantially improved savings. This study emphasises aerogels’ importance in construction and presents an integrated evaluation covering environmental, energy, and economic aspects. The multidisciplinary approach showcased in this study reflects the growing trend in aerogel research to bridge the gap between fundamental science and real-world applications.

Another noteworthy contribution by Patrycja Bober et al. focuses on using polypyrrole aerogels as efficient adsorbents for Cr(VI) ions in aqueous solutions. The adsorption kinetics of Cr(VI) ions onto polypirrole aerogels followed pseudo-first-order and pseudo-second-order models. The development of three-dimensional and porous polypyrrole aerogels highlights the versatility of aerogel materials in addressing environmental challenges.

Furthermore, Mariana Emilia Ghica et al. tackled the challenge of brittleness in silica aerogels by reinforcing them with aramid nanofibers and microfibers. Through reinforcement, they achieved low bulk density and thermal conductivity, improved mechanical resistance, and successful compliance with space conditions. This research represents a significant step forward in enhancing the mechanical properties of silica aerogels for thermal insulation applications.

Ana Dora Rodrigues Pontinha et al. studied the thermomechanical behaviour of various insulation materials, including recycled rubber, aerogels, and new aerogel–rubber composites. The new composite exhibited low thermal conductivity and showed high potential for applications in buildings facing extreme conditions, such as polar regions, very hot climates, and highly humid regions.

The paper by Penghui Li et al. demonstrates the successful preparation of a biomass aerogel with promising adsorption capacity for Congo red and methylene blue, two common dyes found in wastewater. The aerogel’s structure and composition contribute to its excellent swelling properties and uniform pore size, making it a potential candidate for wastewater treatment. The high adsorption capacities for Congo red and methylene blue further support the potential of this biomass aerogel as an effective adsorbent for dye removal.

Jorge Torres-Rodriguez et al. successfully developed a method to produce highly porous bulk PVDF aerogels with precise control of the phase composition without further sample processing or template incorporation. The research demonstrated that the precise control of choice and amount of the components in the precursor solution allowed for excellent control of the crystalline phases, surface morphology, and physicochemical properties in highly porous aerogel monoliths. This study highlighted the roles of ethanol as a nucleation agent and water as a phase-stabiliser compound in the PVDF/DMF/ethanol/water mixture.

Lina Zhang et al. prepared graphene oxide/carbon nanotube/epoxy resin aerogel with 3D cross-linked structures that exhibited excellent compression performance, structural and thermal stability, high hydrophilicity, and microwave absorption. Prepared aerogel recovered from multiple large strain cycles without significant permanent deformation and exhibited a high energy loss coefficient of 65.08%. The minimum reflection loss of this material was −39.60 dB, and the maximum effective absorption bandwidth was 2.48 GHz. 

The main findings of the paper by Chengbin Yu et al. include the excellent thermal energy storage capacity of the cross-linked graphene aerogel-supported PCM composite, the enhanced flexibility of the modified graphene aerogels to reduce volume shrinkage, and the high durability of the cross-linked graphene aerogel to maintain the initial solid state.

Prof. C Pinto Reis’s group published a paper on silica-based aerogels, which demonstrated superior thermal and light-insulating properties compared with pectin-based aerogels, making them more promising for use in non-ionising radiation therapy applications.

Joanta Doneliene et al. reported the cost-effective synthesis of high-porosity TiO_2_ aerogels for solar cell manufacturing. Subcritical drying at 800 mbar vacuum resulted in an amorphous structure, which crystallised into an anatase phase after calcination. In addition, calcination increased apparent and true density, as well as the porosity of aerogels.

The Special Issue includes one review article by Horvat et al., which discusses the challenges in determining the pore size and pore size distribution in aerogels and the importance of these properties for the final application of the materials. The lack of a precise method for pore size distribution or mean pore size in bioaerogels presents a challenge in the future evolution of this field.

The collaborative efforts of the scientific community are evident in these articles, with contributions from researchers exploring different facets of aerogels, from fundamental properties to innovative applications. The studies combined theoretical and experimental data, which is crucial for understanding aerogels and their potential impact on real-life applications. This diverse content presents the current state of the art in aerogel research and lays the groundwork for future advancements in this field. 
